# *Bacillus pumilus* SAFR-032 Genome Revisited: Sequence Update and Re-Annotation

**DOI:** 10.1371/journal.pone.0157331

**Published:** 2016-06-28

**Authors:** Victor G. Stepanov, Madhan R. Tirumalai, Saied Montazari, Aleksandra Checinska, Kasthuri Venkateswaran, George E. Fox

**Affiliations:** 1 Department of Biology and Biochemistry, University of Houston, Houston, Texas, United States of America; 2 Biotechnology and Planetary Protection Group, Jet Propulsion Laboratory, California Institute of Technology, Pasadena, California, United States of America; University of Connecticut, UNITED STATES

## Abstract

*Bacillus pumilus* strain SAFR-032 is a non-pathogenic spore-forming bacterium exhibiting an anomalously high persistence in bactericidal environments. In its dormant state, it is capable of withstanding doses of ultraviolet (UV) radiation or hydrogen peroxide, which are lethal for the vast majority of microorganisms. This unusual resistance profile has made SAFR-032 a reference strain for studies of bacterial spore resistance. The complete genome sequence of *B*. *pumilus* SAFR-032 was published in 2007 early in the genomics era. Since then, the SAFR-032 strain has frequently been used as a source of genetic/genomic information that was regarded as representative of the entire *B*. *pumilus* species group. Recently, our ongoing studies of conservation of gene distribution patterns in the complete genomes of various *B*. *pumilus* strains revealed indications of misassembly in the *B*. *pumilus* SAFR-032 genome. Synteny-driven local genome resequencing confirmed that the original SAFR-032 sequence contained assembly errors associated with long sequence repeats. The genome sequence was corrected according to the new findings. In addition, a significantly improved annotation is now available. Gene orders were compared and portions of the genome arrangement were found to be similar in a wide spectrum of *Bacillus* strains.

## Introduction

The endospore forming species, *Bacillus pumilus*, naturally occurs in the plant root systems of tobacco, pepper, cucumber and tomato, as well as in the leaves of soybean crops [1, 2]. In this context, it can confer systemic protection against several plant diseases [3]. It was therefore unexpected when the strain *B*. *pumilus* SAFR-032 was isolated from a spacecraft assembly facility, and its spores were found to be extremely resistant to radiation, desiccation, and hydrogen peroxide treatment [4, 5].

Subsequently, SAFR-032 spores were found to exhibit high survival rates under exposure to outer space conditions in experiments on board the International Space Station (ISS) [6, 7]. The spaceflight data generated in these studies pointed to the risks of forward contamination of celestial bodies by microbial contaminants carried by landing probes. As a result, *B*. *pumilus* SAFR-032 has been classified as an extreme microorganism according to the planetary protection standards [7], and established as an important reference organism in studies on bacterial spore viability [8–13]. The unique resistance of *B*. *pumilus* SAFR-032 attracted the attention of microbiologists. This interest culminated in the publication of a complete annotated genome sequence of the SAFR-032 strain in 2007 [14]. This preceded by several years the genome of the *B*. *pumilus* type strain, ATCC 7061^T^. Because of its early availability and importance as a model organism, the SAFR-032 genome is often used as a source of genetic information that is considered likely to be representative of the entire *B*. *pumilus* species group [15–22]. The SAFR-032 genome has also been used as a reference for assembly, correction and annotation of closely related genomes [23–25].

In recent years, complete genome sequences were determined for several other *B*. *pumilus* strains [19, 23, 26, 27]. Their comparisons with the SAFR-032 genome revealed discrepancies in gene order, which were possibly due to errors in the original SAFR-032 genome assembly. New studies, described herein, have corrected what were in fact errors. In addition, the SAFR-032 genome annotation was updated to include recently identified genes and correct boundaries of known coding sequences.

## Materials and Methods

### Media, cultivation, and genomic DNA extraction

*B*. *pumilus* SAFR-032 culture growth was initiated from the original stock archived at Jet Propulsion Laboratory (JPL), Pasadena, CA. The cells from the stock were streaked on Tryptic Soy Agar and incubated at 30°C until colonies appeared. A single colony was inoculated into 5 mL of Tryptic Soy Broth, and the culture was grown overnight at 30°C and 200 rpm. The genomic DNA was extracted from 1 mL aliquot using Maxwell 16 DNA/RNA Extraction System (Promega, Madison, WI), and eluted with water [28].

### Primer design

Since the putative sequence misconnections were associated with the repeating genome segments (ribosomal RNA operons or transposase genes), the amplification primers were designed to bind to the sites immediately adjacent to the repeat units. Primer picking was performed using Primer3 v. 2.3.6 [29], primer binding specificity was confirmed using Blast 2.2.29+ [30]. For each questionable junction, 2 primer pairs were selected according to the following criteria: melting temperature 54–56°C, amplicon size not exceeding 10,000 bp, no significant binding outside of the intended priming sites, no significant secondary structure or self-binding. Amplification primers were also used for sequencing the terminal parts of the corresponding amplicons. The internal regions of the rDNA amplicons were partially assessed using universal primers targeting intergenic 16S-23S and 23S-5S spacers. Sequences of all primers used in this study are presented in Table 1.

**Table 1:** Amplification and sequencing primers.

Primer ID	Sequence
Amplification primers	
RRNAO_AC01_fwd	AATTCTTATCTCTTCTTTACAACAAG
RRNAO_AC01_rev	AAGATCATATTCCATTGCTTTATAAC
RRNAO_AC04_fwd	AATGAGAAAGTACTTGAAATCATTAG
RRNAO_AC04_rev	TTAAGAAGAATGGATTTGTAATAACG
RRNAO_BD02_fwd	GAATCATTAGAATTATGAAGGAAGAG
RRNAO_BD02_rev	AGCAATTGATACAAGATAATACTTTG
RRNAO_BD09_fwd	TCATTAGAATTATGAAGGAAGAGAAG
RRNAO_BD09_rev	TTAAATACGTAATACTTTCACCAATC
RRNAO_CB00_fwd	CTGTACTATTGTTGTTTATGTCAGG
RRNAO_CB00_rev	CTTCTATATCTCTTCCTAACACTTG
RRNAO_CB07_fwd	TTACATGTATCTATTAACCCTGTAAC
RRNAO_CB07_rev	TGATATATACATAATCACTACGAGAC
TRANS_L05_fwd	ATATTTATGGTGATACAATACAAGAG
TRANS_L05_rev	TCTTCAATACAGTTCAACATATAATG
TRANS_L09_fwd	GTTCGACTACTTAACGATTAATAAC
TRANS_L10_rev	AAATAATCAAGAAACCTATATCGAAG
TRANS_R01_fwd	GAAATATTTATTCTCGCATTATGAAC
TRANS_R01_rev	CCTATTACATGCTTTCGTTCTTC
TRANS_R03_fwd	AAATATTTATTCTCGCATTATGAACC
TRANS_R05_rev	CTATTACATGCTTTCGTTCTTC
Sequencing primers	
16s_bpum_seq	GCTGGAATCGCTAGTAATCGCGGATCAGCATG
23s_bpum_seq1	CACTAGGGAGTATTTAGCCTTGGGAGATGGTC
23s_bpum_seq2	CGCAAGGAAGTAAGATCCCTGAAAGATGATC

### Amplicon synthesis, purification and sequencing

PCR amplification of the genomic DNA fragments covering questionable *B*. *pumilus* SAFR-032 genome regions was performed using Q5 High Fidelity PCR kit (New England Biolabs, Ipswich, MA). The reaction mixtures contained 0.16 μg of genomic DNA, 1 μM of each amplification primer and 40 μL of Q5 High Fidelity Master Mix in a final volume of 80 μL. After initial incubation at 94°C for 90 sec, the reactions were carried out for 37 cycles at 94°C for 30 sec, 52°C for 30 sec, and 72°C for 5 min. Final extension was performed at 72°C for 10 min. Then, the entire reaction mixtures were separated by electrophoresis on 1% agarose gel containing 0.2 μg/mL ethidium bromide. The bands of relevant size were excised from the gel, and the DNA was isolated from the gel slices on silica spin columns [31]. The purified rDNA amplicons were partially sequenced by the Sanger method at SeqWright, Inc (Houston, TX) using 5 primers for each amplicon: 16s_bpum_seq, 23s_bpum_seq1, 23s_bpum_seq2, and 2 amplification primers. Transposase gene amplicons were sequenced using amplification primers only.

### Genome re-assembly

The *B*. *pumilus* SAFR-032 genome sequence update was performed using Artemis genome viewer v. 16.0.0 [32]. Validation of the re-assembled sequence was done using the Seaview 4.5.3 sequence aligner [33] and Mauve multiple genome aligner, development snapshot 2015-02-13 [34]. Visualization of the aligned genomic fragments utilized Easyfig [35].

### Annotation

The re-assembled new version of the *B*. *pumilus* SAFR-032 genome was annotated using the NCBI Prokaryotic Genome Annotation Pipeline (PGAP) [36] and the RAST pipeline [37]. The results were compared with those of the existing annotated genome (NC_009848.1/CP000813.1). The comparison was conducted by manually aligning the predicted ORFs/genes (including the flanking 20–30 bases upstream) using the BioEdit sequence alignment software [38].

### Genome alignments

Alignments of *B*. *pumilus* genomes were constructed using the Progressive Mauve Aligner [34]. A core alignment included the original *B*. *pumilus* SAFR-032 genome version, CP000813.1, its updated version, CP000813.4, and the following complete genomes: *B*. *pumilus* WP8 (CP010075.1, *de novo* assembly of Illumina MiSeq and PacBio reads), *B*. *pumilus* MTCC B6033 (CP007436.1, *de novo* assembly of PacBio reads), *B*. *pumilus* NJ-M2 (CP012329.1, *de novo* assembly of Illumina and Sanger reads), *B*. *pumilus* NJ-V2 (CP012482.1, *de novo* assembly of Illumina and Sanger reads), *B*. *pumilus* TUAT1 (AP014928.1, *de novo* assembly of 454 and Sanger reads). An extended alignment additionally included the complete genomes of *B*. *pumilus* GR-8 (CP009108.1, a reference-driven assembly of Illumina reads with the *B*. *pumilus* MTCC B6033 genome as a reference), *B*. *pumilus* W3 (CP011150.1, a reference-driven assembly of Illumina reads with the *B*. *pumilus* SAFR-032 genome as a reference), *B*. *pumilus* ku-bf1 (CP014165.1, a reference-driven assembly of Illumina reads with the *B*. *pumilus* W3 genome as a reference). Since some of the genomes were deposited in reverse-complement orientation to a standard layout, they were inverted to bring their orientation to normal. The first position of each genome was set at the first position of *dnaA* gene.

## Results

### Identifying and Correcting Sequencing Problems

**Fig 1:**
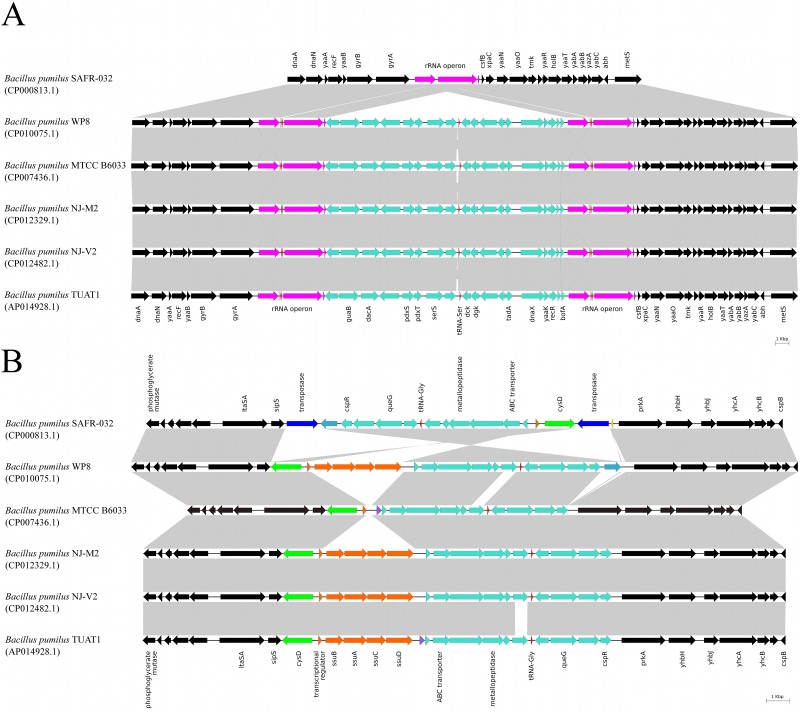
Synteny violations between the complete genomes of *B*. *pumilus* SAFR-032 (CP000813.1) and other *B*. *pumilus* strains. Only *de novo* assembled genomes of *B*. *pumilus* strains were considered. Multiple genome alignments were performed with the Progressive Mauve Aligner [34]. The aligned segments of interest were further evaluated with BLASTN and visualized using Easyfig [35]. Genes within homologous syntenic blocks are colored with the same color except transposase, rRNA and tRNA genes, which are colored in blue, magenta and red, respectively, regardless of their belonging. Perfectly syntenic gene clusters present in all aligned genomes in the same orientation are shown in black. (A) *dnaA* (BPUM_0001)—*metS* (BPUM_0022) genome fragment. (B) *gpmB* (BPUM_0834)—*cspB* (BPUM_0862) genome fragment.

Six *de novo* assembled complete genomes of *B*. *pumilus* strains SAFR-032 (CP000813.1), WP8 (CP010075.1), MTCC B6033 (CP007436.1), NJ-M2 (CP012329.1), NJ-V2 (CP012482.1), and TUAT1 (AP014928.1) were aligned using the Mauve aligner to investigate conservation of *B*. *pumilus* genome layout. The original assembly of the SAFR-032 genome deviates from the consensus gene order near the origin of bacterial chromosome replication (Fig 1A). A conserved genome fragment encompassing 19 protein-encoding genes, 1 stand-alone tRNA gene and 1 rRNA operon was found displaced. This fragment is 522 kbp downstream of its location in the other genomes as related to the position of chromosomal replication initiation protein *dnaA*. Another SAFR-032 genome segment containing 13 protein-encoding genes and 1 tRNA gene, and flanked by transposase coding sequences was found to be inverted as compared to the genomes of other strains (Fig 1B).

**Fig 2:**
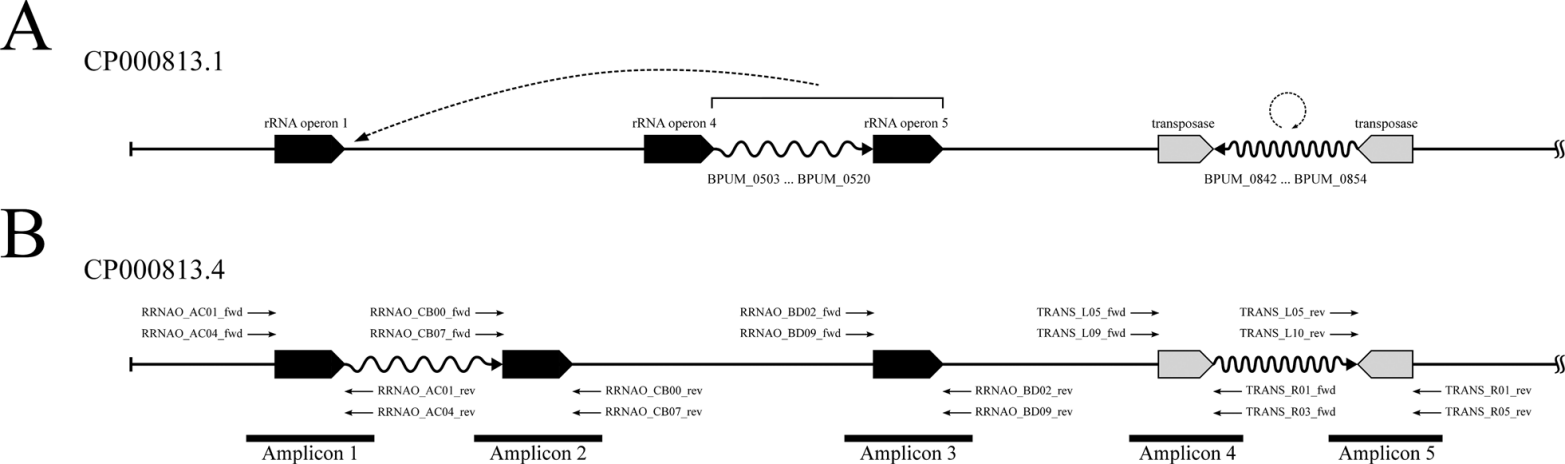
**Proposed *B*. *pumilus* SAFR-032 genome rearrangements (A) and scheme of their validation (B).** Not to scale. rRNA operons and transposase genes are shown as black- and grey-filled rectangular arrows, respectively. The problematic fragments between sequence repeats are shown as waved arrows. The proposed sequence rearrangements are shown as dashed arrows. Relative positions of amplification primers are shown by thin straight arrows. The span of PCR amplicons is marked by thick black lines. Sequence start corresponds to the first codon of *dnaA* gene.

Both problematic fragments are flanked with long sequence repeats, rRNA operons in one case and transposase genes in the other. This makes it look like genome assembly errors were caused by incorrect contig joining over repeat regions. Better agreement with other *B*. *pumilus* strains can be achieved by moving a stretch of genes BPUM_0503-BPUM_0520 and the ribosomal operon 5 (BPUM_r0013, BPUM_r0013, BPUM_r0015) immediately downstream of the ribosomal operon 1 (BPUM_r0001, BPUM_r0002, BPUM_r0003), and by inverting a genome segment harboring genes from BPUM_0842 to BPUM_054 (Fig 2A). Validity of this rearrangement has been confirmed by selective PCR amplification of the affected SAFR-032 genome regions followed by dye dideoxy terminator sequencing of the amplicons (Fig 2B).

### Comparison of New PGAP Annotation with Original Annotation and Resolution of Differences

Although the SAFR-032 genome rearrangement did not affect the existing gene boundaries, the genome sequence was re-annotated to bring it to the most up-to-date state. The primary annotation was performed with NCBI PGAP [36] and the results were compared with the original (CP000813.1) annotation. A surprisingly large number of differences were found and significant effort was required to resolve them.

To begin with, the automated PGAP annotation omitted 170 genes as well as all non-coding RNAs that have been present in the original genome annotation. These genes were checked for their veracity and those that were found to be genuine ORFs based on their homology to other genomes in the NCBI databases, were manually incorporated into the new annotation (S1 Table). Known non-coding RNAs were also added back. The revised version of the annotated *B*. *pumilus* SAFR-032 genome sequence was deposited in Genbank under accession number CP000813.4.

In addition, a total of 385 ORFs were found to differ between the new PGAP annotation and the original (CP000813.1) annotation in terms of the predicted location of the start codon (S2 Table). These questionable gene boundaries were examined using the RAST pipeline [37]. In 197 cases RAST supported the new PGAP ORF coordinates. In 118 cases RAST placed the start codon position as it was in CP000813.1, and thus the earlier annotation was retained. Finally, in 70 cases RAST did not support either annotation. Hands on visual examination made it clear that the problematic ORFs do not have recognizable ribosome-binding sites in front of the candidate start codons. In the absence of experimental data, this makes the prediction of ORF start positions in these 70 cases unreliable, and does not allow a clear choice between several available alternatives. Therefore in the final updated annotation (CP000813.4), for consistency reasons, the ORFs with ambiguous coordinates were left as annotated by PGAP.

In the PGAP annotation, 58 entries were marked as pseudogenes (S3 Table). The two annotations agreed on 35 pseudogenes. The new PGAP version had 23 extra pseudogenes, 22 of which were previously regarded as valid genes, and 1 was newly identified. After examination of sequence alignments with close homologs, it was concluded that nine of these are indeed genuine pseudogenes with either in-frame stop codons or deletion of a substantial part of the coding sequence. The remaining fourteen examples rather correspond to properly expressed genes as it follows from homology analysis. Thus, only 44 pseudogenes were retained in the final CP000813.4 annotation.

### BPUM_2060: A Special Case

BPUM_2060 was annotated by PGAP as a pseudogene of a "bifunctional GTP cyclohydrolase II/3,4-dihydroxy-2-butanone 4-phosphate synthase" (*ribA*). This gene is a part of the four gene operon *ribTHAE* that codes for enzymes involved in the riboflavin biosynthetic pathway [39]. The pseudogene status was assigned to BPUM_2060 because it maps to only the N-terminal half of the reference protein (WP_006636466.1, product of *ribA* gene from *Bacillus sonorensis*), which makes BPUM_2060 look like a truncated version of the reference. However, it appears that in some *Bacillus* species, GTP cyclohydrolase II and 3,4-dihydroxy-2-butanone 4-phosphate synthase are two distinct polypeptides encoded by separate genes. In *B*. *pumilus* SAFR-032, BPUM_2060 gene encodes 3,4-dihydroxy-2-butanone-4-phosphate synthase while two other genes, BPUM_0700 and BPUM_3531, each encodes GTP cyclohydrolase II. Besides *B*. *pumilus* strains, the same situation exists in *B*. *safensis*, *B*. *altitudinis*, *B*. *xiamenensis*, *B*. *stratosphericus*, *B*. *isronensis*, *B*. *simplex*, and *B*. *butanolivorans*. It can also be seen outside of the *Bacillus* genus, for example, in *Planococcus donghaensis*, *P*. *halocryophilus*, *P*. *antarcticus*, *Solibacillus silvestris*, *Lysinibacillus fusiformis*, *L*. *sphaericus*, *L*. *varians*, *Paenibacillus polymyxa*, *Kurthia massiliensis*, *K*. *huakuii*, and *Oceanobacillus manasiensis*. Therefore, BPUM_2060 was re-annotated as a valid gene. This example demonstrates that an improperly selected reference may cause an annotation artifact. Clearly, in the present day scenario of rapidly evolving sequencing technologies, automated annotation of genomes is likely to be error-prone, and manual curation is absolutely necessary to maintain better standards of quality of a genome and its various components.

### Final Updated Annotation

Overall, the updated *B*. *pumilus* SAFR-032 genome annotation contains 3710 valid protein-encoding genes, 44 likely pseudogenes, 21 rRNA genes grouped in 7 rRNA operons, 72 tRNA genes, one tmRNA, 4 other non-coding RNA genes, and 16 riboswitches. The updated annotation includes 38 genes and 2 riboswitches that were not present in the original CP000813.1 genome annotation (S4 Table). Three of the newly identified genes, BPUM_03795, BPUM_04085, and BPUM_04135, are transposases, which brings a total number of mobile genetic elements in *B*. *pumilus* SAFR-032 genome to 20. Another two genes, BPUM_04115 (coding for the “capsular biosynthesis protein CpsH”) and BPUM_04155 (coding for a “spore coat protein”) with probable roles in cellular structural integrity and sporulation, respectively, may be an addition to the candidate genes involved in the unusual resistances exhibited by *B*. *pumilus* SAFR-032 [10, 11]. Among the rest, seven genes are predicted to be involved in transport and secretion, two genes encode regulatory proteins, three genes encode enzymes, and five genes are predicted to encode stable cellular RNAs (three tRNAs, tmRNA, and 6S RNA). The final revised version of the annotated *B*. *pumilus* SAFR-032 genome sequence was deposited in Genbank under accession number CP000813.4.

## Discussion

High-throughput shotgun sequencing methods generate a steady stream of genomic sequences filling public databases. Currently, the majority of deposited prokaryotic genomes have draft status. Thus, they represent a collection of contigs or scaffolds rather than a single circularly closed chromosome. While being acceptable for mutation mapping, strain typing, and metabolic pathways identification, the draft genome assemblies are not sufficient for studying large-scale genome architecture, which is needed for better understanding of genome function and evolution. This creates a demand for completed genome sequences, even though the assembly finishing is costly and time-consuming. The major obstacle on the way towards a complete genome is posed by long sequence repeats, which may cause assembly errors because of ambiguities in contig order. The present study demonstrates that such errors can be revealed using multiple alignments of closely related genomes.

All *de novo* assembled complete *B*. *pumilus* genomes shows absolute conservation of gene order in the region immediately downstream of the replication origin, between *dnaA* and *metS* (*metG*) genes. This observed genetic layout is well preserved far beyond the *B*. *pumilus* strains. With minor modifications it can be seen in *B*. *subtilis*, *B*. *mojavensis*, *B*. *atrophaeus*, *B*. *amyloliquefaciens*, *B*. *licheniformis*, *B*. *megaterium*, *B*. *endophyticus*, *B*. *smithii*, *B*. *cytotoxicus*, in the entire *B*. *cereus* group of species, and throughout *Geobacillus* and *Anoxybacillus* genera (S1 Fig). Thus, the deviation of *B*. *pumilus* SAFR-032 genome sequence from such a pronounced trend strongly indicated a high possibility of an assembly error. Direct sequencing of the problematic locus confirmed the presence of an error. The corrected SAFR-032 sequence exhibits perfect agreement with the consensus gene distribution pattern near the replication origin.

The second problematic segment in the SAFR-032 genome covering genes from *gpmB* (BPUM_0834) to *cspB* (BPUM_0862) is significantly less conserved. Among six *de novo* assembled *B*. *pumilus* genomes, only strains WP8, NJ-M2, NJ-V2, and TUAT1 contain *ssuBACD* operon in this location while in SAFR-032 and MTCC B6033 it is missing. The SAFR-032 gene BPUM_0842 encoding an YncM-like protein maps to the QR42_04470 gene in the aligned fragment of the WP8 genome but is absent in the corresponding genomic regions of other strains. Two identical transposase genes are present only in the SAFR-032 genome fragment and flank the gene stretch from BPUM_0842 to BPUM_0854, which was inverted in the earlier *B*. *pumilus* SAFR-032 genome version, CP000813.1, as related to other *B*. *pumilus* genomes. The relatively low local conservation of the gene order has made the indications of sequence misassembly look weaker than in the previous case. Yet, the reversal of a 11,000 bp-long genome fragment was regarded as a sufficiently substantial deviation from the consensus gene pattern to justify its re-evaluation.

## Conclusions

**Fig 3:**
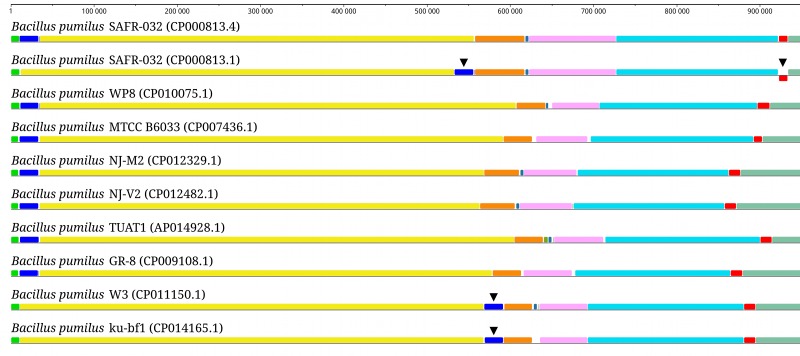
Comparative genome analysis of complete *B*. *pumilus* genomes. Multiple genome alignments were performed with Progressive Mauve Aligner [34]. Related segments have the same color in all aligned genomes. Inverted segments are shown below a genome's center line. Only the first 950,000 bp of each genome are shown for the entire alignment (S2 Fig). Both *de novo* and reference-driven genome assemblies are presented. The problematic genome fragments are marked with black triangles.

Possible assembly errors in the previously published *B*. *pumilus* SAFR-032 genome were evaluated and corrected. To accommodate these changes, re-annotation was required. Despite the fact no coding sequences were changed this led to significant changes in the annotation that required considerable effort to evaluate. This points to continuing difficulties in automated annotation and highlights the importance of being cautious when relying on it. The immediate consequences of the SAFR-032 genome sequence update reflect the organism’s long-standing role as a reference for closely related species. The SAFR-032 genome was previously used as an assembly template in reference-driven assembly of *B*. *pumilus* W3 genome, which in its own turn was used as a reference for *B*. *pumilus* ku-bf1 genome assembly. Therefore, it is not surprising that both W3 and ku-bf1 genomes exhibit the same deviant layout that was seen in the CP000813.1 version of the SAFR-032 genome (Fig 3 and S2 Fig). As in the case of *B*. *pumilus* SAFR-032, these two genomes may need re-evaluation. Furthermore, all intergenomic synteny studies that have involved *B*. *pumilus* SAFR-032 strain may need to be critically revised.

## Supporting Information

S1 Fig Synteny analysis of gene cluster *dnaA*—*metS* (*metG*) in family *Bacillaceae*. Red dashed outline shows the limits within which the synteny is generally preserved. (A) BLASTN alignments of *dnaA*—*metS* (*metG*) segments visualized with Easyfig. Protein-coding sequences, rRNA and tRNA genes are colored in cyan, magenta and red, respectively. (B) Fragment of PATRIC phylogenetic tree of order *Bacillales*. The tree is based on genome-wide analysis of homologous protein groups.
(TIF)

S2 Fig Comparative genome analysis of complete *B*. *pumilus* genomes. Multiple genome alignments were performed with Progressive Mauve Aligner. Related segments are identically colored in all aligned genomes, and are connected with a line of the same color through the entire alignment. Inverted segments are shown below a genome's center line.
(TIF)

S1 Table List of genes in the original *B*. *pumilus* SAFR-032 genome annotation (CP000813.1), which were not included by NCBI PGAP into the new annotation (CP000813.4). (XLSX)

S2 Table List of genes with ambiguous start codons in the *B*. *pumilus* SAFR-032 genome annotation. (XLSX)

S3 Table List of pseudogenes in the new *B*. *pumilus* SAFR-032 genome annotation (CP000813.4). (XLSX)

S4 Table List of new genes and pseudogenes identified by NCBI PGAP in the new *B*. *pumilus* SAFR-032 genome annotation (CP000813.4). (XLSX)
