# Supplementary figures and images for: Bacillus pumilus SAFR-032 Genome Revisited: Sequence Update and Re-Annotation
Source: PLoS One. 2016 Jun 28;11(6):e0157331. doi: 10.1371/journal.pone.0157331 (PMC4924849; doi:10.1371/journal.pone.0157331)

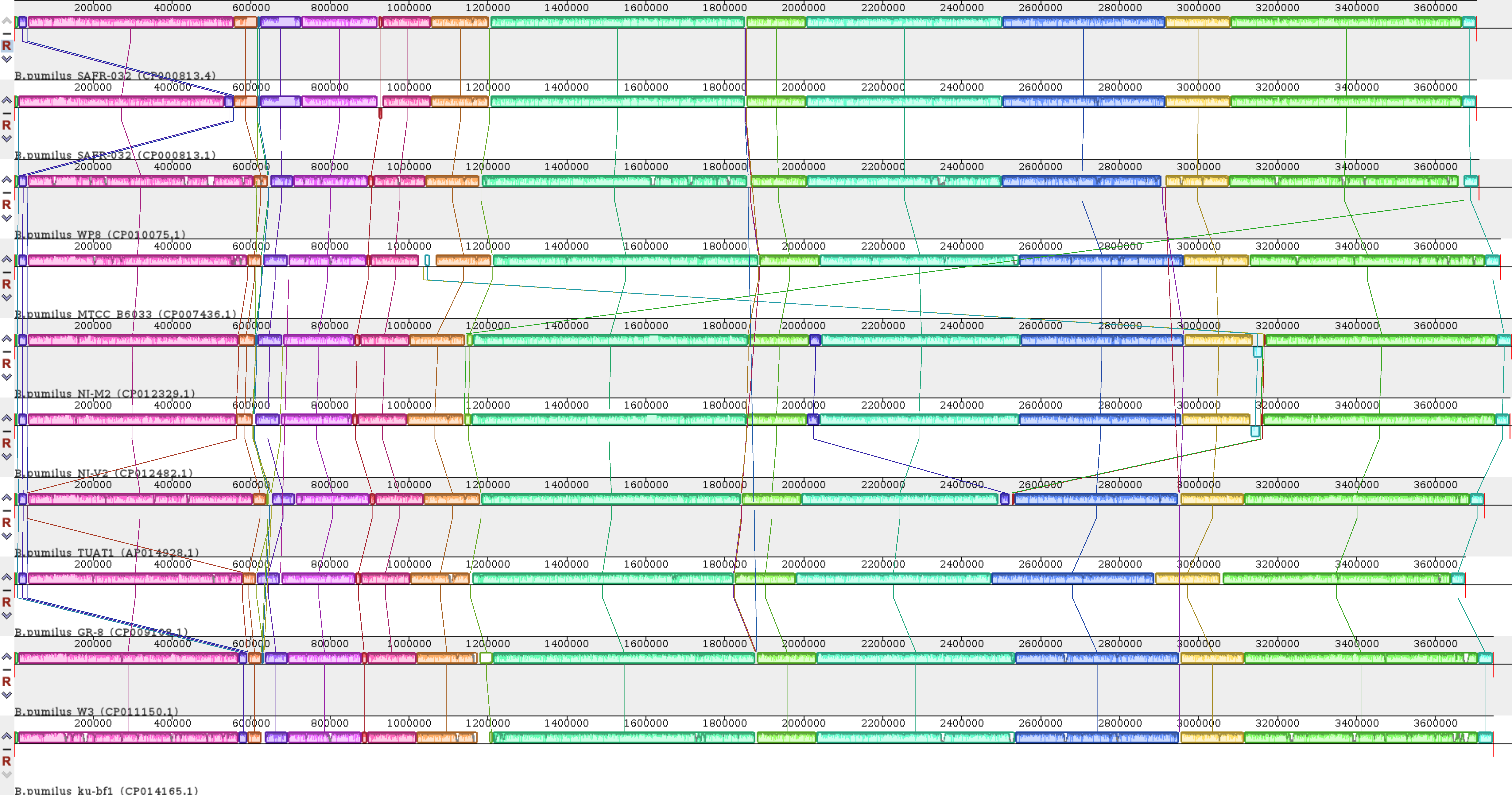

Supplement: S2 Fig — Multiple genome alignments were performed with Progressive Mauve Aligner. Related segments are identically colored in all aligned genomes, and are connected with a line of the same color through the entire alignment. Inverted segments are shown below a genome's center line. (TIF) [file pone.0157331.s002.tif]
